# Williams Syndrome As a Model for Elucidation of the Pathway Genes – the Brain – Cognitive Functions: Genetics and Epigenetics

**Published:** 2014

**Authors:** E. A. Nikitina, A. V. Medvedeva, G. A. Zakharov, E. V. Savvateeva-Popova

**Affiliations:** Pavlov Institute of Physiology, Russian Academy of Sciences, nab. Makarova, 6, 199034, St. Petersburg, Russia; Herzen State Pedagogical University, nab. r. Moyki, 48, 191186, St. Petersburg, Russia; Saint Petersburg State University, Universitetskaya nab., 8-9, 199034, St. Petersburg, Russia

**Keywords:** Williams syndrome, LIMK1, non-coding RNAs, Drosophila

## Abstract

Genomic diseases or syndromes with multiple manifestations arise spontaneously and unpredictably as a result of contiguous deletions and duplications generated by unequal recombination in chromosomal regions with a specific architecture. The Williams syndrome is believed to be one of the most attractive models for linking genes, the brain, behavior and cognitive functions. It is a neurogenetic disorder resulting from a 1.5 Mb deletion at 7q11.23 which covers more than 20 genes; the hemizigosity of these genes leads to multiple manifestations, with the behavioral ones comprising three distinct domains: 1) visuo-spatial orientation; 2) verbal and linguistic defect; and 3) hypersocialisation. The shortest observed deletion leads to hemizigosity in only two genes: *eln *and *limk1*. Therefore, the first gene is supposed to be responsible for cardiovascular pathology; and the second one, for cognitive pathology. Since cognitive pathology diminishes with a patient’s age, the original idea of the crucial role of genes straightforwardly determining the brain’s morphology and behavior was substituted by ideas of the brain’s plasticity and the necessity of finding epigenetic factors that affect brain development and the functions manifested as behavioral changes. Recently, non-coding microRNAs (miRs) began to be considered as the main players in these epigenetic events. This review tackles the following problems: is it possible to develop relatively simple model systems to analyze the contribution of both a single gene and the consequences of its epigenetic regulation in the formation of the Williams syndrome’s cognitive phenotype? Is it possible to use* Drosophila *as a simple model system?

## WILLIAM S SYNDROME AND DI SCO VERY OF GENOTY PE-PHENOTY PE CORRELATION S

**Fig. 1:**
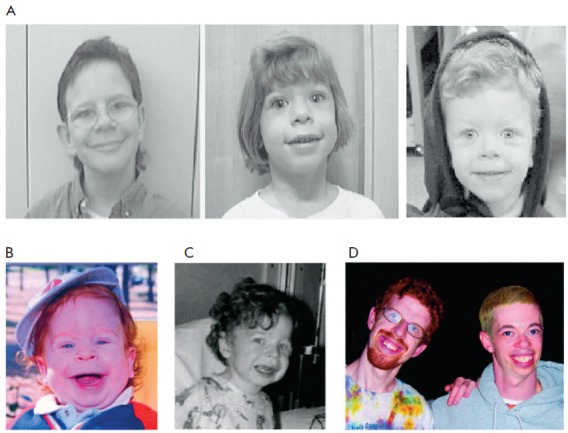
Distinctive facial appearance of persons with WBS (A) [5]. Young child with WBS at the age of 15 months (B) and 3 years (C). Note subtle characteristic facial features, including wide mouth, chubby cheeks, long philtrum, small nose, and delicate chin. The same patient is shown in Figs. 1B, 1C, and 1D (left; 21 years); another individual with WBS aged 28 years is shown in Fig. 1D (right) [3]

In 1961, J.C.P Williams, summarizing his observations in four patients, suggested that “the simultaneous occurrence of supravalvular stenosis and typical physical and mental characteristics correspond to a new syndrome that was not previously reported” [1]. Soon after, in 1962, A.J. Beuren described another 11 similar patients. All of them displayed specific facial features and mental retardation, along with supravalvular aortic stenosis [2]. Since then, the eponym “Williams– Beuren syndrome (WBS)” has become a common name for this set of symptoms, which is also often known as the Williams syndrome. This syndrome occurs due to a deletion spanning 1,500 kb at the q11.23 region of human chromosome 7. The specific architecture of this region predisposes it to unequal recombination. The deletion covers about 20 genes; the hemizygosity of these genes has multiple effects: a specific, “elfin” facial appearance (*Fig. 1*), developmental disorders, a variety of cardiovascular diseases, neurological abnormalities and cognitive features, hypersocialisation, and musical talent [3]. This combination of unusual properties has been intriguing and has attracted neuroscientists as an opportunity to understand the modular principle of mental abilities and social behavior structure, reflecting the features of brain development. Over the past 20 years, the Williams syndrome has been considered to be one of the most attractive models that directly link genes, the brain, behavior, and cognitive functions [4, 5].

**Fig. 2:**
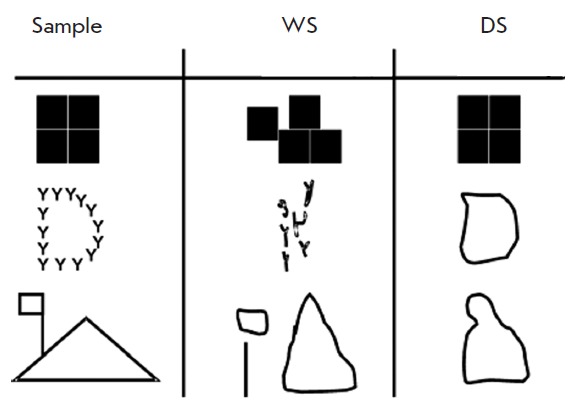
Visuo-spatial deficiency in WBS patients [8]. Left: images the patients were asked to draw. Middle: WS – WBS patients reproduce only disconnected elements, ignoring the global form. Right: DS – Down syndrome patients (age- and IQ-matched) reproduce only the global forms. This figure demonstrates a featural perception in WBS patients compared with a holistic perception in DS patients

Neurological abnormalities include hyperactivity, as well as deficiency of motor coordination and gait [6, 7]. Cognitive manifestations are very specific; for this reason, they are used to diagnose WBS in young children, along with neurological symptoms. The first manifestation is a pronounced deficiency in visuo-spatial orientation; patients cannot reproduce the shape of an object in standard tests, but they can reflect all its parts (*Fig. 2*) [8].

Visuo-spatial construction is the ability to perceive an object or a picture as a set of parts and then use these parts to build a replica (i.e., an exact copy or reproduction of what a person saw). People use visuospatial construction when they draw, button up a shirt, make their bed, create models of sailing ships and aircrafts, piece together LEGO building blocks, or furniture purchased unassembled at an IKEA store. Visuospatial construction is very important in daily life; for this reason, it is considered to be the central cognitive ability. Therefore, measuring this ability is an integral part of any complete testing of the mental abilities of an individual.

**Fig. 3:**
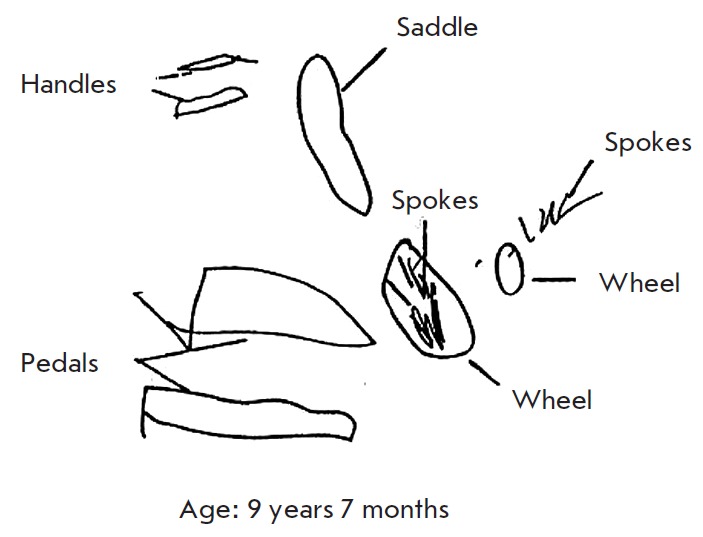
Drawings of a bicycle by a girl aged 9 years 7 months with the Williams syndrome [10]

**Fig. 4:**
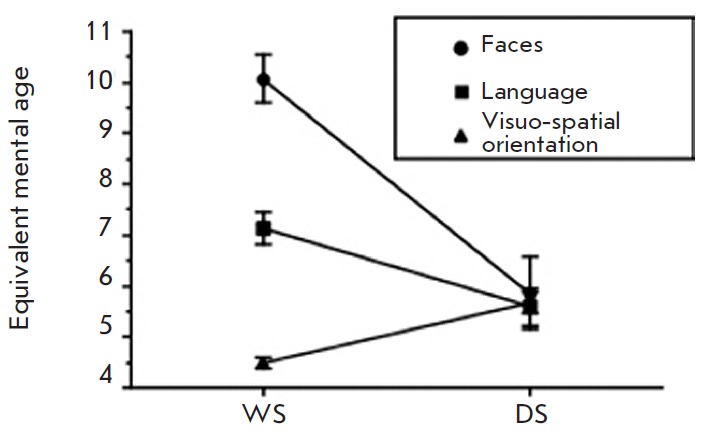
Three distinct domains of cognition in Williams syndrome (WS) patients and Down syndrome (DS) patients (age- and IQ-matched) [5]. Labels: Faces – fixation on faces; Language – linguistic abilities; Visuo-spatial – visuo-spatial orientation. Patients with DS demonstrate equally low levels of all the parameters of cognitive ability as expected in mental deficiency. Patients with WBS show pronounced defects of visuo-spatial orientation, have reduced linguistic abilities, and show extreme hypersocialization (fixing on the faces of onlookers)

Japanese children with visuo-spatial construction deficiency have difficulty when learning hieroglyphic writing [9]. When requested to draw a bicycle, children draw an image including separate, clearly reproduced and signed items: handles, a saddle, pedals, wheels, and spokes (*Fig. 3*) [10]. Furthermore, many patients have neither binocular vision nor normal perception of space and its depth. For this reason, they face daily challenges when walking or playing games on uneven surfaces. The second manifestation is the immensely high level of evaluative vocabulary in prejudice of grammar, when plenty of emotional interjections, sighs and accents serve as a “hook” to attract and hold the attention of onlookers. This is related to the third manifestation, hypersocialisation: i.e., the need to establish contact with any persons, including strangers, unusually high sympathy for them, and the desire to make everyone happy. This manifestation is currently considered to be one of the leading cognitive features; regardless of the proposed test, patients always closely examine the faces of the experimenters, ignoring the matter of the test [11]. Thus, cognitive impairment in the Williams syndrome patients includes a triad of manifestations: 1) a pronounced deficiency of visuo-spatial orientation; 2) intermediate verbal-linguistic defect, varying depending on the complexity of the language culture; and 3) unusually intense gaze with fixation on faces (*Fig. 4*).

The life inconveniences caused by manifestations of this triad are compensated for by the high musical talent. Every patient perfectly plays a musical instrument or sings. Unusually, high thirst for music allows them to perceive and reproduce the phenomena of the world in musical, rather than visual, images. Thus, magnetic resonance imaging of the brain shows activation of the visual cortex upon presentation of music or any sound stimuli in patients with WBS, unlike their healthy peers [12]. On the one hand, the phenomenon of WBS redefines the old stereotypes. Is it true that everything should be perfect in a person? Is it important for us whether Paganini, Beethoven, and Bach could draw well? On the other hand, clear and discrete cognitive manifestations constantly inspire to associate them with a certain gene, falling within deletion critical for WBS. Let us recall the mechanism of genomic disease occurrence: i.e., deletion-duplication syndromes.

## NON -ALLELIC RECOMBINATION PRODUCING THE WILLIAM S SYNDROME

Genomic diseases or syndromes with multiple manifestations occur spontaneously and unpredictably (sporadically) as a result of extensive deletions and duplications generated due to unequal recombination in chromosomal regions with a specific architecture. These are the Williams syndrome in 7q11.23 [3], Smith- Magenis syndrome in 17p11.2 [13], DiGeorge syndrome in 22q11.2 [14], Prader-Willi and Angelman syndrome in 15q11-q13 [15], duplication syndrome (17) (p11.2p11.2), and syndromes with Y-chromosome deletions [16]. A high frequency of such structural rearrangements of the genome, significantly exceeding the frequency of occurrence of a disease due to mutations of a single gene, drew the attention of clinicians and led to the appearance of the concept of “genomic diseases.”

**Fig. 5:**
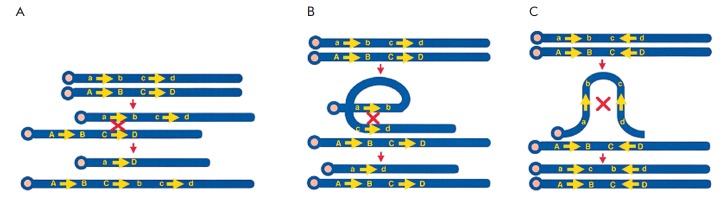
Genomic rearrangements resulting from recombination between duplicons [17]. (A) – interchromosomal recombination between direct repeats results in deletion and/or duplication; (B) – intrachromosomal recombination between direct repeats (in one homolog) results in deletion; (C) – intrachromosomal recombination between inverted repeats results in an inversion. Repeated sequences are depicted by yellow arrows with the orientation indicated by the direction of the arrows. Recombination is shown by red X. Upper- and lowercase letters (e.g., A and a) refer to a unique flanking sequence in different allelic states

In most deletion-duplication syndromes, the reconstructed chromosomal segment is flanked by large (usually 10–500 kb), highly homologous low copy repeat sequences (LCR ), for which the recombination occurs. Due to the fact that in this case recombination involves homologous, but not allelic sequences, the term “non-allelic homologous recombination” (NAHR) appeared. As a result of NAHR between direct repeats in the same chromosome duplications and deletions occur, and reverse orientation results in inversion (*Fig. 5*). NAHR between different chromosomes leads to the formation of translocations [17].

**Fig. 6:**
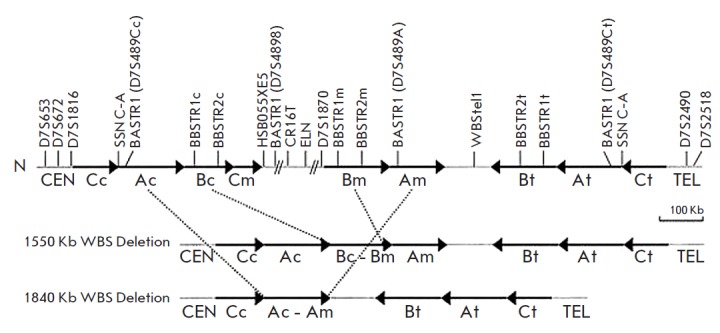
Schematic representation of the 7q11.23 genomic region in normal chromosomes (N) and chromosomes with WBS deletions [18]. A, B, and C blocks of centromeric (c), medial (m), and telomeric (t) LCRs are represented by black arrows that indicate their relative orientation. The single-copy regions between and outside the LCRs are shown as gray lines. The limits of typical deletion (1.55 Mb) and rarer deletion (1.84 Mb) found in our WBS patients are indicated by dotted lines

The most detailed study of the role of LCR in genomic diseases was conducted using WBS as an example [18]. WBS deletion is flanked by three LCR s (centromeric, telomeric, and medial); each of them consists of blocks A, B and C [19]. Blocks of centromeric and medial repeats are arranged in the same orientation, but in different order, while the telomeric segment is in the same order, but in opposite orientation (*Fig. 6*). Block B consists of three genes in the medial location (Bm) (GTF21, NC F1, GTF21RD2), alleged pseudogenes in the centromeric region (Bc) (GTF21P1, NC F1P1, GTF21RD2P1), and telomeric region (Bt) (GTF21P2, NC F1P2, GTF21RD2P2). In most patients (95%), the deletion of 1,550 kb occurred as a result of nonhomologous crossover between the centromeric (Bc, or telomeric Bt in the case of inversion in parents) and medial blocks of repeats. A more extended deletion (1,840 kb) is caused by the exchange between the Ac and Am blocks, registered in 5 % of cases. The preferred localization of exchanges in block B is obvious. Breaks can occur anywhere in the repeat; nevertheless, there is a tendency toward the formation of clusters of breaks in the proximal region of Bc/Bm blocks, where, apparently, a hot spot sized 12 kb is localized, which is 11.4 % of the whole sequence of the block. This area accounts for 67% of the recombinations.

Polymorphism in the organization of the LCR flanking the deletion allows one to suggest the possibility of other genomic rearrangements. Indeed, 30% of parents of children with WBS have an inversion spanning the entire WBS interval [20]. It is believed that WBS deletion occurs due to non-allelic intrachromosomal or interchromosomal recombination; in this case, the identity of the repeats plays a crucial role [21]. However, in the case of inversion in the parents [18], nonhomologous crossover occurs during the first meiotic division and affects the last 38 kb of the Bt block, which are absent in the Bc block. The positional preference of NAHR exchanges may be due to the additional architectural features of these areas. It is important to note for further consideration that in some cases palindromes capable of forming a hairpin are located close to the hot spot [22].

**Genes localized within the deletion**

The following genes are located within the deletion (*Fig. 6*). Most of them (two-thirds) encode proteins that to some extent organize the space in the nucleus or cytoplasm. Thus, some of them encode transcription factors (WBSCR 9/WSTF, WS-bHLH, WBSCR 11/GTF2IRD1) which form the core protein compartments, and others are involved in the reorganization of cytoskeletal and membrane-bound structures (LIMK1, STX1A, CYLN2, TBL2, CLDN4 / CPTER 1, CLDN3/CPTER 2). A brief description of some genes is presented below.

*frizzled-9 (fzd9) *encodes the Frizzled-9 protein, similar to the *Drosophila *wnt receptor. This gene is involved in the development of the hippocampus in mice. Hemizygous state of only this gene leads to severe cognitive impairment, including defects in the neuroanatomy of the hippocampus and, as a consequence, impairment of memory and spatial orientation [23], which is very similar to the manifestations of the complex effect of deletion in the Williams syndrome.

*stx1a *encodes STX1A, syntaxin 1A, a syntaxin family member, specific for the brain protein with a molecular weight of 35 kDa. It is required for the release of a neurotransmitter from the synaptic vesicle. Syntaxin 1A interacts with synaptotagmin and other proteins of synaptosomes [24]; therefore, an assumption was made about the role of syntaxin gene hemizygosity in the neurological symptoms of WBS [25].

*eln *encodes tropoelastin, a component of elastic fibers. This gene is located in the middle of the deletion region; thus, it is a deletion marker. Hemizygosity of the tropoelastin gene leads to the formation of stenosis, thinning of the arterial walls, and underdevelopment of muscles. Apparently, it is responsible for the specific elfic appearance of WBS patients [26].

*cyln2 *encodes the cytoplasmic linker protein CLIP- 115, which connects the endosomes to the growing microtubules through specific binding to their ends. Thus, CLIP-115 is involved in the reorganization of microtubules and effects their interaction with various cellular structures. CLIP-115 is expressed predominantly in the brain and localizes in the lamellar body of dendrites.

*wbscr11/gtf21rd1 *encodes the transcription factor GTF2I containing a characteristic helix-loop-helix motif and TFII-I calcium channel regulator with a high and ubiquitous expression.

*limk1 *encodes non-receptor serine-threonine protein kinase, the key enzyme in actin remodeling [22, 27].

**Fig. 7:**
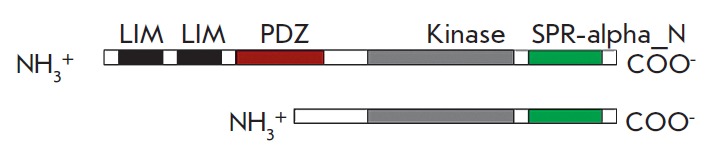
Domain structure of LIMK1. Top – C isoform; bottom –D isoform, both the LIM domains and PDZ domain are absent

**Fig. 8:**
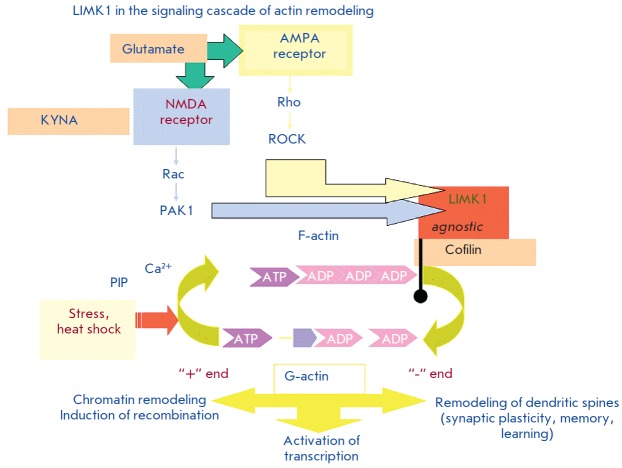
Scheme of the signaling cascade of actin remodeling

The LIMK1 molecule consists of four domains: the kinase domain, as well as two LIM and one PDZ domains (*Fig. 7*). Deletion of the LIM and PDZ that are responsible for interaction with other proteins increases the kinase activity of the molecule, which is indicative of the regulatory function of these domains [28]. LIMK1 interacts through the LIM-domain with a variety of proteins, including protein kinase C, the cytoplasmic domain of the transmembrane neuregulin ligand [29].*Fig. 8* illustrates the signaling pathway of actin remodeling.

LIMK1 activity is regulated by members of the Rho GTPase family; namely, Rho, Rac, and Cdc42, through protein kinase ROCK, p21-activated kinase (PAK), i.e. PAK1 and PAK4, respectively. These kinases phosphorylate Thr508 in a loop of the kinase domain of LIMK1, leading to its activation [30]. Cofilin acts as a target for the LIMK1 involved in actin depolymerization when attached to the sharp end of the actin filament. When cofilin is phosphorylated by LIMK1, it is inhibited and disconnected from the actin filaments. Thus, LIMK1 controls actin dynamics by cofilin switching from the active to the inactive state [31]. Reorganization of the actin cytoskeleton is involved in neuron movement and neurite growth. Actin remodeling is required for the emergence and modification of dendritic spines, which form most synaptic connections in the hippocampus and other brain areas, and thus mediate learning and retention of the memory trace. In addition, the transcription factors CRE B and Nurr1 act as a physiological substrate for LIMK1. LIMK1 also phosphorylates myelin basic proteins and histones *in vitro *[29].

**Fig. 9:**
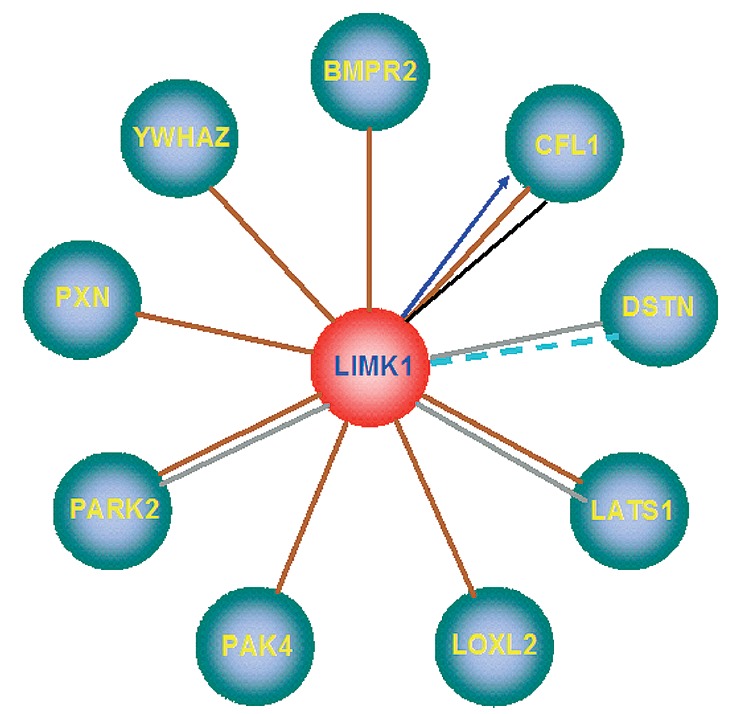
Interaction of LIMK1 with other proteins. BMPR2 – Bone morphogenetic protein receptor, type II (serine/ threonine kinase); CFL1 – Cofilin 1 (non-muscle); DSTN – Destrin (actin depolymerizing factor); LATS1 – LATS, large tumor suppressor, homolog 1 (*Drosophila*); LOXL2 – Lysyl oxidase-like 2 (*Drosophila*); PAK4 – P21 protein (Cdc42/Rac)-activated kinase 4; PARK2 – parkin 2 (*Drosophila*); PXN – Paxillin; YWHAZ – Tyrosine 3-monooxygenase/ tryptophan 5-monooxygenase activation protein, zeta polypeptide Drosophila gene *leonardo *(affects olfactory learning)

The partner genes that produce the proteins that interact with LIMK1 have been identified (*Fig. 9*).

It is assumed that hemizygosity of this gene is one of the factors that determine the appearance of defects of visuo-spatial behavior in WBS patients. The list of genes affected by the deletion continues to grow and currently includes 28 genes. This list is adequately represented in the survey [8], which purports to establish genotype-phenotype correlations.

A deletion of minimum length leads to hemizygosity for only two genes, *eln *and *limk1* (*Fig. 6*). As a result of studying their manifestations, the former gene was considered to be the crucial one in the genesis of cardiovascular pathology; the latter gene, of cognitive pathology. This viewpoint was based on comparative characteristics of the expression of both genes in the brain: expression of *eln *was very low, whereas the expression level of *limk1 *was very high and reached a maximum in the cerebral cortex [32]. However, although this study has conclusively proven the role of hemizygosity of the *limk1 *gene in the formation of visuo-spatial orientation defects, the other one could not confirm this role [33].

It should be recalled that, although the deletion resulting in the Williams syndrome occurs with a frequency exceeding the frequency of mutations in a single gene due to a higher frequency of unequal recombination, each study includes not that many patients (so far there have been five of them in St. Petersburg). As a rule, the deletion boundaries (breakpoints in the chromosome) are not identified when confirming the deletions; the spontaneity and unpredictability of deletions prevents an intrafamilial analysis. However, this is possible in rare cases, because there are families with identical deletions [34].

Thus, in five families with supravalvular arterial stenosis a small deletion was revealed; it led to the Williams syndrome in all of them and affected the *limk1* gene, but not *fkbp6 *or *gtf2i*. All carriers of this deletion demonstrated defects of visuo-spatial orientation, but not mental retardation; therefore, the role in the genesis of the former was left to LIMK1, whereas the GTF2I transcription factor was suspected to be involved in the genesis of mental retardation [ 35].

However, the known limitations of studies on human objects necessitated a recourse to animal models. Obviously, the first attempts to establish the role of a specific gene in the Williams syndrome manifestations were made on mice. This object provides an easy way to obtain carriers of null mutations, as well as hypomorphic and point mutations and deletions involving many genes in the region of interest [36]. Moreover, unlike humans, who can have only one affected child with a random chromosome deletion obtained from mother or father, it is possible to obtain numerous offspring of mice with the same genetic disorders.

**Fig. 10:**
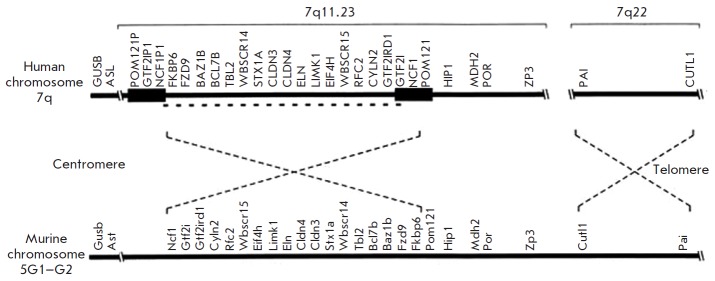
Order of genes within the WBS region in a human and a mouse. The dotted line shows an inversion of each type with respect to telomere and centromere [19]

It should be noted that the order of the genes within the deletion is evolutionarily conserved and is the same in mice as in humans (*Fig. 10*). However, the region with breakpoints in identical flanking sequences is inverted with respect to the genomic map of the similar region in humans and contains no low copy repeats [18].

Data accumulated over the past 10 years indicate that hemizygosity in similar genes in humans and mice do not always lead to similar manifestations. Nevertheless, it was conclusively proven that the formation of cognitive and behavioral manifestations involves two genes that control the cytoskeleton function by regulating actin dynamics (*limk1*) [37] and the microtubule network (*clip2*) [38], similarly to those in humans. Hemizygosity at the *cyln2 *gene (*clip2*) in mice leads to damage typical of WBS, moderate developmental disorders, abnormalities in motor coordination, brain morphology, and hippocampal dysfunction.

However, very little attention has been paid to the analysis of the participation of this (as well as all other investigated genes of mice) in the control of visuo-spatial orientation [36]. This process is also known as spatial memory, which is responsible for the hippocampus. It can be tested in mice in a Morris water maze. Mice placed in the maze learn to escape quickly and correctly to the invisible underwater platform, localizing it by means of “signals” of the environment; i.e., signs specially painted on the walls of the room around the maze or randomly located objects (switches, etc.). Knockdown of only one *limk1 *gene leads to strong visuo-spatial dysfunction due to physiological and morphological hippocampal dysfunction [37]. The former manifests itself as impairment of synaptic plasticity (long-lasting potentiation), induced by NMDA receptors defectiveness; the latter manifests itself as a change in the morphology of the dendritic spines of hippocampal pyramidal cells, which is indicative of the direct function of LIMK1, the key enzyme in actin remodeling that determines the morphology of spines.

Apparently, cognitive disorders can be induced both by hemizygosity of LIMK1 itself and violation of the interaction with partner proteins of LIMK1 due to hemizygosity (*Fig. 9*), such as the product of the *park2* autosomal recessive gene (*parkin*). Let us recall that this gene is responsible for the early onset of Parkinson’s disease; it produces E3 ubiquitin ligase. A recent analysis of the WBS deletion in mice has led to the discovery of a previously unknown fact that the *trim50* gene encodes E3 ubiquitin ligase [39].

## FROM GENETICS TO EPIGENETIC

**Fig. 11:**
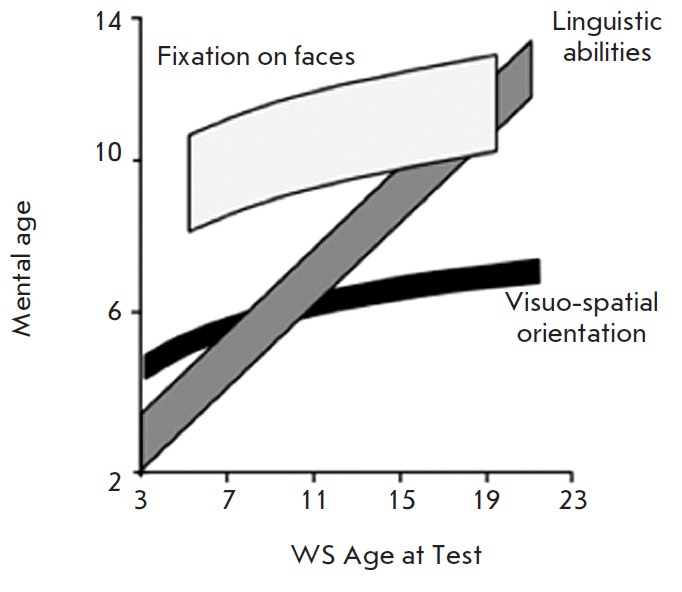
Changes in three main cognitive features vs. age of WBS patients [5]

**Fig. 12:**
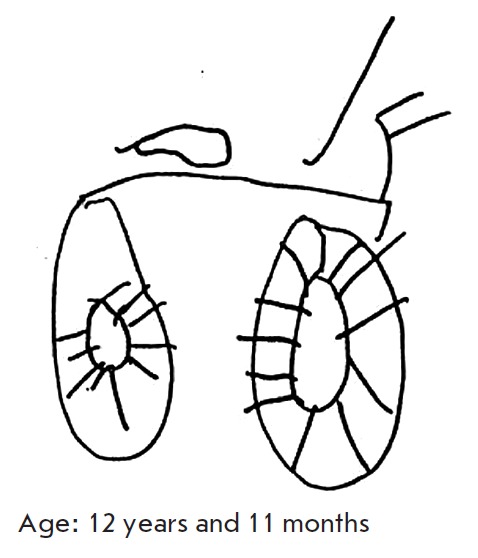
Drawings of a bicycle by a girl aged 12 years and 11 months with the Williams syndrome [10]

It would seem that the functional role of the LIMK1 enzyme and the gene encoding it in the formation of the Williams syndrome’s pathology has been proved. However, there is now data on a long-term analysis of cognitive manifestations in the same patients who have grown from small children to teenagers and young adults. It has been established [9, 40] that both visual and linguistic defects smoothen with age. Perception and display of the whole shape, rather than separate details, become possible; verbal intelligence increases, while evaluative and emotional coloring are retained (*Fig. 11*). For example, at the age of 9 years, a child who was asked to draw a picture of a bicycle drew signed details; i.e., he perceived only parts of the whole (*Fig. 3*); however, at the age of 12, the same child was already able to synthesize the whole object and its parts (*Fig. 12*).

Therefore, the original understanding of the exclusive role of genes directly determining the path to brain morphology and behavior has evolved to a suggestion of the relevance of searching for the epigenetic factors of brain plasticity that affect its development and functions (reflected in changing behavior) [40].

This has led to a different interpretation of the seemingly contradictory data. Let us recall that in most cases a limited number of patients of all ages (from toddlers to 14- and 19-year-olds) are being studied. Thus, a clear picture of the genetic determination at the beginning of life (one gene – one enzyme – behavioral manifestation) is superimposed on the different epigenetic changes in the gene’s action, depending on the experienced social (family and school) stress and individual experience (learning, conditioned reflex). Therefore, research into the Williams syndrome, supported by nuclear magnetic resonance data and modern brain-imaging to identify some particular areas of the brain that are activated upon testing for the behavioral triad, has veered toward looking at the individual development of children upon interaction with the environment [5].

It is the individual development of an organism, including humans [41, 42], that is considered in the tideway of the transactional analysis; i.e., transactions (interactions) between the genotype and the environment. It is assumed that biobehavioral systems are capable of adaptive self-organization and self-stabilization through conditioned reflexes to environmental signals [43].

According to modern concepts, such transactions lead to epigenetic changes. They occur not only due to the already known phenomena, such as methylation of gene promoters and acetylation of histones, but also due to a new phenomenon: the regulation of gene activity by small non-coding (nc) RN As.

As regards the first aspect, the epigenetics of changes in gene activity with age becomes an independent field of research [44]. It is believed that the genes are “turned on” when DNA is unmethylated and histones are acetylated, and, conversely, genes are “turned off” when the DNA is methylated and histones are nonacetylated. This is a dynamic process that depends on age, diet, and stress [45].

The second aspect is new and unusual. Thus, we are witnessing growth in research into a direction contradicting the established molecular genetics paradigm. It has been established that only 1.2% of mammalian genes encode protein products, while the rest of the genome generates various classes of ncRN As. For this reason, a new paradigm has appeared [46]. According to this paradigm, the known classes of ncRN As and those that are yet to be discovered allow for the regulation of the expression of the genes encoding proteins in normal and pathological conditions.

This interaction between the two “worlds,” i.e. RN A and proteins, is the basis for a flexible relationship between the genes and the environment, which is essential for the functioning of the nervous system. Moreover, ncRN A is a device for communication between the digital information in the nucleic acids of cell nucleus and the analogous information in cellular proteins [47, 48].

**Fig. 13:**
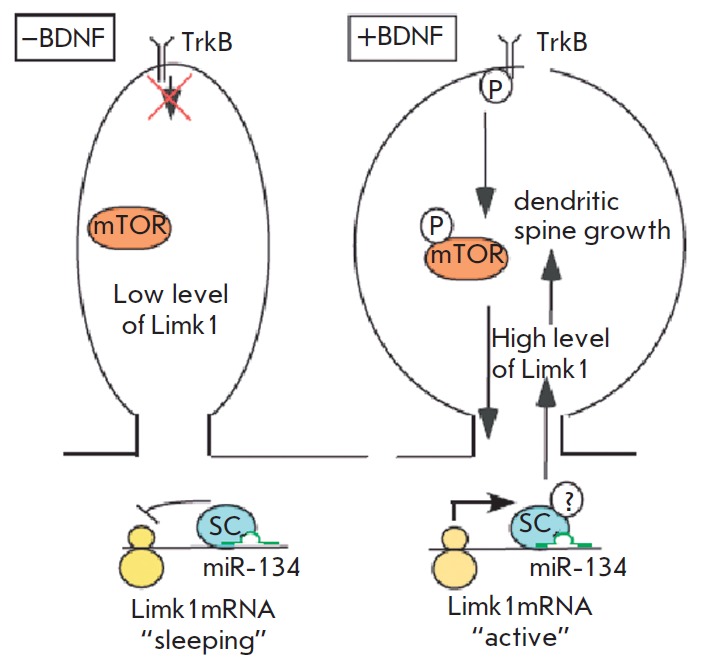
miR-134 in the regulation of local translation of LIMK1 [49]. In the absence of BDNF, translation of LIMK1 is blocked by miR-134 through a silencing complex (SC) leading to a reduction in dendritic spines. In the presence of BDNF, translation of LIMK1 and dendritic spine growth are activated

Functioning of ncRN As, which are predominantly present in the nervous system, provides synaptic plasticity, the molecular foundation of memory formation. While short-term memory (up to 3 h), i.e. memory about events that have just occurred, is based on a modification (generally phosphorylation) of pre-existing proteins, the medium-term memory (2-8 h) depends on the synthesis of new proteins based on pre-existing messenger RN A (mRN A), i.e. local translation in dendrites and synapses distant from the nucleus of the nerve cell, regulated by miRN As. They participate in the formation of “silent” miRN A-mRN A complexes convenient for transportation from the nucleus to the dendrite, which requires some transport machinery (the actintubulin microtubules of dendrites). Dendritic transport of many mRN As may be regulated via the interaction of the PDZ-domains of LIMK1 with the tubulin of microtubules [29]. It is worth recalling that development of the cognitive and behavioral manifestations in the Williams syndrome involves two genes that control cytoskeleton functions by regulating actin dynamics (*limk1*) [37] and the microtubule network (*clip2*) [38]. This group of mRN As includes templates for a rapid local synthesis of glutamate receptor subunits, in particular NMDA and GluR, postsynaptic density (PSD) proteins, transcription factors, and components of a signal cascade of actin remodeling (LIMK1, cofilin). The widely cited example [49] reports interaction between miR-134 miRN As and the mRN A of the LIMK1 protein, the key enzyme in actin remodeling, to create a “silent” complex and local translation of mRN A encoding the LIMK1 protein in dendrites in response to neuronal activity (*Fig. 13*). miR-134 is partially complementary to the 3’-untranslated region of the mRN A of LIMK1 (3’UTR ).

microRN A is the most intensively studied class of ncRN As sized 20-30 nucleotides in length and operating according to the principle of RN A interference. Heterochromatin is a source of small RN As. It is the key factor in epigenetic regulation of gene expression, chromosome behavior, nervous system functions in health and disease, as well as evolutionary transformations [50]. Chromatin modifications are coordinated with the activation of transcription cascades in synaptic plasticity and directly related to the CRE B-dependent signaling pathways.

New models are required to explore this new phenomenon. This raises a number of questions.

Is it possible to find and design fairly simple systems to analyze the contribution of both a single gene and the consequences of its epigenetic regulation in the formation of a cognitive profile in Williams syndrome’s patients, abstracting from the complex epigenetic factors of individual brain development from infancy to adolescence (in humans) or postnatal development (in mice)? Is it possible to use drosophila for this purpose? ;

## Drosophila melanogaster AS A PLAUSIBLE MODEL TO EXPLORE THE PATHWAYS GENES – BRAIN – MIND : GENETICS AND EPIGENETICS

On the one hand, the functions of the so-called pathological human genes are often identified by the nature of the manifestations of mutations in the same gene of *Drosophila*, if this gene has the same sequence as that of the human gene. On the other hand, all the genes concentrated in one critical region in the mammalian genome (let us recall that the *frizzled-9 *gene within the Williams syndrome deletion was the first to be described in Drosophila) are scattered on different chromosomes in *Drosophila*. Despite the other path of evolutionary organization, i.e. different localization of genes that are linked in mammals, this approach to the analysis of the function of a specific gene in the genesis of the Williams syndrome is possible under the following conditions:

1) the mutations in a given gene must be known, and the hemizygosity of this gene lead to the manifestation of a mutant phenotype in Drosophila;

2) the architecture of the chromosomal region where the Drosophila gene is localized may be a predisposition to the occurrence of chromosomal rearrangements by unequal recombination;

3) increased frequency of recombinations is registered in the region of gene localization, which might lead to spontaneous generation of deletions or other rearrangements; and

4) wild-type lines are characterized by a polymorphism specific to this region.

We have found and described *agnostic D. melanogaster* locus carrying the gene encoding the LIMK1 protein, which meets all these criteria.

**The *agnostic *locus**

The *agnostic *locus was found in the 11B region of the X chromosome of *D. melanogaster *during the targeted screening of temperature-sensitive (*ts*) mutations induced by ethylmethane sulfonate (EMS) in the *Canton- S (CS) *line, which can affect the activity of the enzymes of cAMP synthesis and degradation [51].

The *agn^ts3^*mutant at this locus displays an unusually high activity of Ca^2+^/calmodulin-dependent phosphodiesterase Pde1 [52].

A molecular genetic study of the locus revealed a 21 kb DNA fragment within the region of deletions. EcoRI fragments of 7, 5. and 9 kb within this region were subcloned, and their terminal nucleotide sequences were identified.

**Fig. 14:**
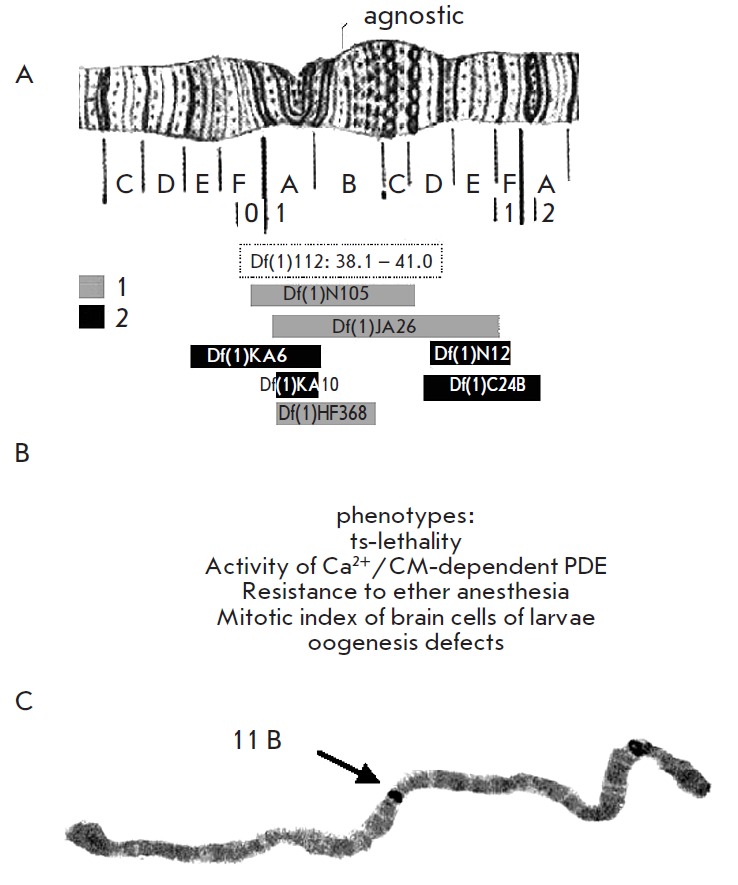
Localization of the *agnostic *locus within the X chromosome [54]. (a) Deletion mapping. The length of rectangles (except for *Df(1)112 *microdeletion, for which the limits of recombinational mapping are shown) represents the length of the deletions in the X chromosome, resulting (1) and not resulting (2) in phenotypic manifestations of mutations (b) at the *agnostic *locus; (c) *in situ* hybridization of P-element DNA with the polytene chromosomes of the P-insertional mutant *P40*

We used Southern blot hybridization to demonstrate that the wild-type lines *Canton-S (CS), Berlin *and* Oregon-R (Or-R) *are characterized by a pronounced polymorphism precisely in this region. The results of a bioinformatic analysis allowed us to arrange these fragments within the AE003489 segment of the 11B region of the X chromosome of Drosophila (*Fig. 14*).

It turned out that this area, which falls within both the known deletion *Df(1)368 *and the narrow deletion* Df(1)112 *we have obtained, contains the gene encoding the LIMK1 protein, which is homologous for a huge number of species, including humans.

The results of our bioinformatic analysis revealed the homology of *agnostic *locus, mainly the 5 kb EcoRIfragment, with three known forms of LIM kinases from different vertebrate species [53, 54].

**Fig. 15:**
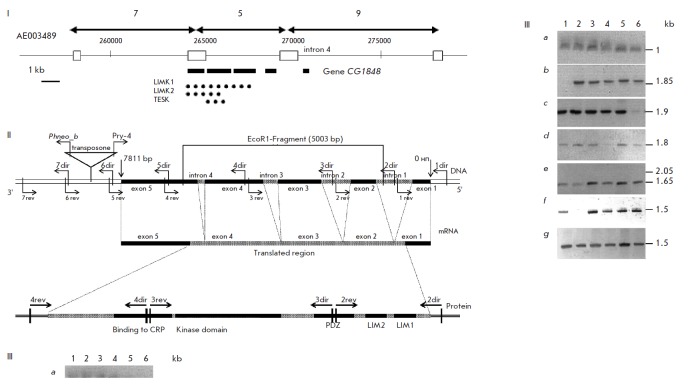
Schematic representation of the exon-intron structure of the *CG1848 *gene (Lim-kinase) with five exons and four introns (I), sites of primer binding (II), and PCR results (III) [55]. I. Localization of sequenced ends (solid arrows) of the genomic 7-, 5-, and 9-kb EcoRI fragments in the published sequence AE003489 (open boxes). Numerals designate ordinal numbers of nucleotides of the AE003489 fragment; solid boxes, exons of the *Drosophila CG1848* gene for LIMK1; solid dots, amino acid sequences of the* CG1848 *gene product with regions of homology with LIMK2 and TESK of various species shown. The figure is oriented according to the centromere position on the right; the direction of the gene transcription is from the centromere to the telomere. 1 – *Oregon-R*, 2 – *agnosticts3*, 3 – *Canton-S Hall*, 4 – *Df(1)112*, 5 – *Canton-S*, 6 – *Berlin*. Letters denote the regions limited by primers: a - 1dir-1rev; b - 2dir-2rev; c -3dir-3rev; d- 4dir-4rev; e – 5dir-5rev; f – 6dir-6rev; g – 7dir-7rev

The occurrence of *agn^ts3^*mutant phenotypes was observed under conditions of *(Df ( 1 ) 112/CS)* hemizygosity (e.g. high levels of activity of Ca^2+^/calmodulin- dependent kinase Pde1 and nonhomologous chromosome pairing). It was shown [53, 54] that the region of the *agnostic *gene contains repetitive sequences both within (repeat of two LIM domains), and around, the gene. The gene is flanked by extensive AT-rich repeats (The National Center for Biotechnology Information, NC BI). Therefore, the high polymorphism of the spontaneous and mutant alleles of this gene *(Fig. 15)*, shown by D.A. Molotkov using PCR [55], is probably due to non-homologous crossover.

Thus, the *agnostic *gene can play the role of a genetic reserve of polymorphism and be a convenient model of genomic disorders, such as the Williams syndrome, because of its structure and environment. In the region of the *agnostic *gene localization, crossover frequency is threefold higher compared with that in the control. The highest numbers of double exchanges, negative interference, and nonreciprocal complementary crossover classes are observed under thermal action (29°C) at the end of the embryonic or at the beginning of the larval stage of development, rather than at the stage of premeiotic DNA synthesis (late larva III- chrysalis).

This proves that the mutation does not affect the crossover itself, but rather its background, changing the pairing features of chromosomes.

A southern blot analysis of the genomic DNA reveals an additional Sall fragment of 11 kb in *agn^ts3^*mutants. Therefore, it has been suggested that the frequency of exchanges increases due to unequal crossover, resulting in the occurrence of a Sall fragment in the *agn^ts3^* mutant, presumably due to insertions or duplication.

Indeed, PCR mapping of the *agn^ts3^*mutant in the regulatory region of the *limk1 *gene revealed an insertion of 1.7 kb, located approximately 1 kb below the 3’UTR .

The insertion site is consistent with the AT-rich region, which is capable of forming a hairpin in the single- stranded conformation and structure of club cross in a double-stranded conformation identified in the database. We assume that this anomalous structure can serve as a preferred spot of insertion of natural transposon and is also capable of producing miRN A with a complex secondary structure and properties similar to those of miR-134 during its transcription. The possibility of the participation of these miRN As allows one to explain many aspects of the regulation of the gene’s action [55].

The *agnostic *gene displays the following characteristic features:

1. Immunofluorescence studies of the distribution of LIMK1, the key enzyme of actin remodeling the signal cascade, in the brain areas of Drosophila revealed that it preferentially localizes in the central complex of the brain and in the visual system. Mutational damage in the *limk1 *gene (at *agn^ts3^*) leads to a sharp increase in LIMK1 activity in all brain areas. The same effect in wild-type *Canton-S *flies is caused by thermal exposure.

2. The hemizygous state of the *limk1 *gene in Drosophila leads to a change in LIMK1 distribution in the brain areas, similarly to that in the Williams syndrome in humans. The enzyme is localized exclusively in the visual system and loses its dependence on the thermal effect.

3. The immunofluorescence study of the distribution of LIMK1 and cofilin phosphorylated by the enzyme (pcofilin) in the cells of the salivary glands of Drosophila larvae revealed their predominantly cytoplasmic localization in wild-type flies. A heat shock causes the transfer of components of the signaling cascade of actin remodeling into the nucleus and leads to a sharp increase in the activity of LIMK1 and p-cofilin. Mutational damage in the LIMK1 gene (*agn^ts3^*mutation) increases the content and activity of LIMK1: this effect is disappears under the influence of a heat shock.

4. Mutational damage to the *limk1 *gene (EMS- and P-insertional mutations at *agnostic *locus) affects the pairing properties of chromosomes in Drosophila. Thus, the frequency of formation of ectopic contacts in the regions of intercalary heterochromatin in salivary gland polytene chromosomes dramatically increases the hemizygosity of the gene, identically to that in hu mans with the Williams syndrome, and results in the expression of the mutant phenotype.

5. The *agnostic *gene is involved in the mechanisms of homologous synapsis of chromosomes, resulting in a sharp decrease in the asynapsis frequency in the *agnts3* line and abnormalities in the distribution of long and short asynapses along the chromosome. This is indicative of differences in the localization of chromosomal arms in the nucleus with respect to each other in the wild-type and agn^ts3^, i.e. different ways of three-dimensional spatial organization of the nucleus.

6. Mutational damage to the signaling cascade of actin remodeling leads to the formation of amyloid aggregates in the brain of imago and in the larval tissues of all agn^ts3^ samples. The incidence of aggregates is reduced to the standard level after a heat shock. This correlates with learning ability and memory formation. Overexpression of LIMK1 in mutants is accompanied by a significant reduction in the learning ability and medium-term memory revealed in the conditionedreflex suppression of courtship in males. The method is based on stimuli that are natural to the sexual behavior of Drosophila [56].

## CONCLUSIONS

A high frequency of deletion-duplication syndromes, including the Williams syndrome, leads to the emergence of the concept of “genomic diseases,” which allows one to link genes, the brain, behavior, and the cognitive function. Clarity and discretization of cognitive manifestations made it possible to identify the key gene responsible for the cognitive component of the syndrome, i.e. the *limk1 *gene. A study of the occurrence of intragenomic reserves of the syndrome (i.e. clusters of repetitive sequences), the distribution of these regions over zones with different conformations in the chromosome, and creation of a specific organization of the nucleus, in which the spatial convergence of functionally and structurally related regions of chromosomes is achieved, was required. This was the motivation behind designing animal models. In particular, the study of *agnostic *locus for LIMK1 of Drosophila revealed the presence of repetitive sequences in the region of the gene. Mutant expression of this gene is associated with changes in the pairing properties of the chromosome and three-dimensional organization of the nucleus, which is an epigenetic derivative of mutational damage.

In the language of genetics, the following chain of events emerges when analyzing the mutant and spontaneous variants of *agnostic *locus: external signal – activation of LIMK1 – cofilin phosphorylation – state of actin – normal cognitive abilities or abnormal memory loss, accompanied by the formation of congophilic (amyloid) deposits.

Thus, we can assume that the *agn^ts3^*mutant line is a model of the Williams syndrome in Drosophila. The revealed relation between mutational damage to the* limk1 *gene, change in the expression and activity of LIMK1, presence of amyloid-like inclusions and cognitive impairments allow one to be able to apply this model in the study of both neurodegenerative and genomic diseases. The availability of natural polymorphic variants in the *limk1 *gene allows one to use them as a tool when studying neurodegenerative diseases, which in most cases occur spontaneously under the influence of adverse environmental factors. The possibility of using the described tools is the subject matter of special experimental studies being conducted in our laboratory [57].

## Abbreviations

WBS: Williams-Beuren syndrome
LCR: low copy repeat
NAHR: non-allelic homologous recombination
miRs: microRNAs
